# Action for Increasing Diversity, Market Access, and Capacity in Oncology Registration Trials—Is Africa the Answer? Report From a Satellite Session of the Accelerating Anti-Cancer Agent Development and Validation Workshop

**DOI:** 10.1200/GO.22.00117

**Published:** 2022-06-17

**Authors:** Darya Kizub, Cathyryne K. Manner, Katy Graef, Bello Abubakar, Jackson Orem, Folakemi Odedina, Mojisola Christianah Adeyeye, Gertrude Nakigudde, Kassa Ayalew, Chitkala Kalidas, Herbert Kim Lyerly, Thea Norman, Lola Fashoyin-Aje, Jamie Freedman, Jennifer Dent, Bill Cance, Julie Gralow

**Affiliations:** ^1^ University of Texas MD Anderson Research Center, Houston, TX; ^2^ BIO Ventures for Global Health, Seattle, WA; ^3^ National Hospital, Abuja, Nigeria; ^4^ African Organization for Research and Training in Cancer (AORTIC), Rondebosch, South Africa; ^5^ Uganda Cancer Institute, Kampala, Uganda; ^6^ Mayo Clinic, Rochester, MN; ^7^ National Agency for Food and Drug Administration and Control (NAFDAC), Lagos, Nigeria; ^8^ Uganda Women's Cancer Support Organization (UWOCASO), Kampala, Uganda; ^9^ United States Food and Drug Administration, Silver Spring, MD; ^10^ Bayer, Pittsburgh, PA; ^11^ Duke University, Durham, NC; ^12^ Bill & Melinda Gates Foundation, Seattle, WA; ^13^ Genentech, South San Francisco, CA; ^14^ American Cancer Society, Atlanta, GA; ^15^ American Society of Clinical Oncology, Alexandria, VA

## Abstract

Patients of African ancestry are not well-represented in cancer clinical trials despite bearing a disproportionate share of mortality both in United States and Africa. We describe key stakeholder perspectives and priorities related to bringing early-stage cancer clinical trials to Africa and outline essential action steps. Increasing Diversity, Market Access, and Capacity in Oncology Registration Trials—Is Africa the Answer? satellite session was organized at 2021 Accelerating Anti-Cancer Agent Development and Validation Workshop. Panelists included representatives of African Organization for Research and Training in Cancer, Uganda Cancer Institute, Uganda Women's Cancer Support Organization, BIO Ventures for Global Health, Bill & Melinda Gates Foundation, the US Food and Drug Administration, Nigeria's National Agency for Food and Drug Administration and Control, Bayer, and Genentech, with moderators from ASCO and American Cancer Society. Key discussion themes and resulting action steps were agreed upon by all participants. Panelists agreed that increasing diversity in cancer clinical trials by including African patients is key to ensuring novel drugs are safe and effective across populations. They underscored the importance of equity in clinical trial access for patients in Africa. Panelists discussed their values related to access and barriers to opening clinical trials in Africa and described innovative solutions from their work aimed at overcoming these obstacles. Multisectoral collaboration efforts that allow leveraging of limited resources and result in sustainable capacity building and mutually beneficial long-term partnerships were discussed as key to outlined action steps. The panel discussion resulted in valuable insights about key stakeholder values and priorities related to bringing early-stage clinical trials to Africa, as well as specific actions for each stakeholder group.

## PURPOSE AND METHODS

Noncommunicable diseases, including cancer, are the leading cause of mortality in all regions of the world. Cancer outcomes vary by race and ethnicity because of differences in environmental exposures and cancer incidence,^1^ tumor biology,^2-6^ treatment side effects,^7,8^ and socioeconomic determinants of health.^9^ In Africa, cancer prevalence and mortality are projected to rise substantially by 2040^10^ because of increased longevity and adoption of more sedentary Western lifestyles and diets.

**CONTEXT**

**Key Objective**
We describe key stakeholder perspectives and priorities related to bringing early-stage cancer clinical trials to Africa and outline essential action steps.
**Knowledge Generated**
Priorities of patient advocacy representatives, oncology research organizations, pharmaceutical companies, and regulatory agencies included increasing both diversity and equity in early-stage cancer clinical trials to improve generalizability of results and build long-term sustainable collaborations with partners in Africa. Such multisectoral collaboration allowed for leveraging of limited resources and was key to outlined action steps and increasing access to early-stage clinical trials in Africa.
**Relevance**
The discussion among key stakeholders resulted in valuable insights about key stakeholder values and priorities related to bringing early-stage cancer clinical trials to Africa, as well as specific agreed-upon actions for each stakeholder group to bring registration clinical trials to patients.

**FIG 1:**
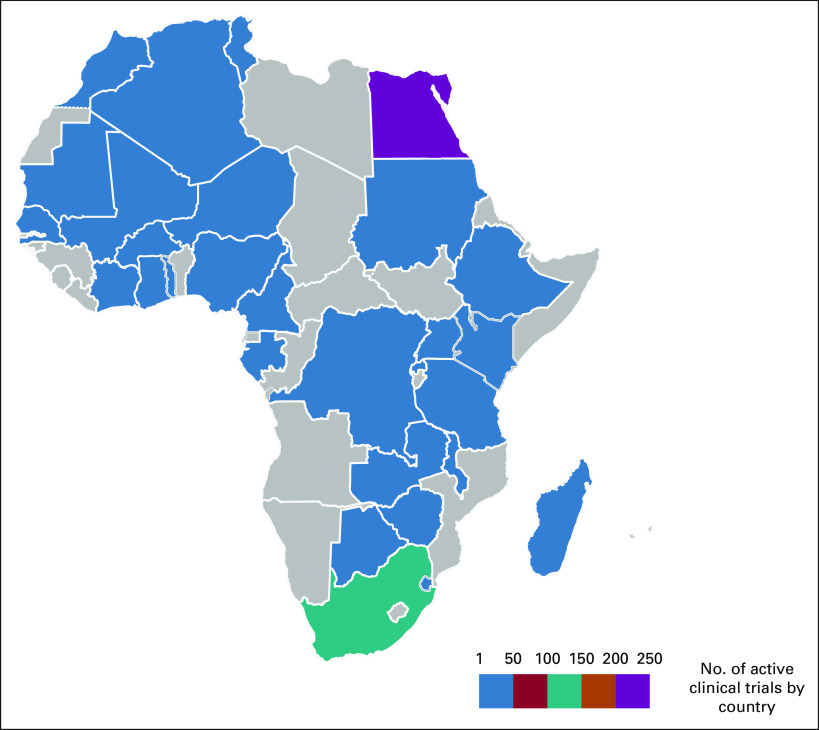
Heat map of current cancer clinical trials in Africa.

The availability of cancer clinical trials reflects a country's clinical research capacity and is likely associated with the provision of evidence-based cancer care and improved patient outcomes.^11^ Although countries in Africa account for a very small percentage of worldwide cancer clinical trials,^12,13^ including 1,376 trials total and 444 active trials (Table 1, Fig 1, on the basis of data from ClinicalTrials.gov), the trials that have been performed have resulted in practice-changing insights.^14-16^ These studies are particularly important given that, compared with patients in Europe and North America, patients in Africa respond to treatment differently because of cancer biology,^3,5^ nutritional status, and HIV coinfection.^17^

**TABLE 1:**
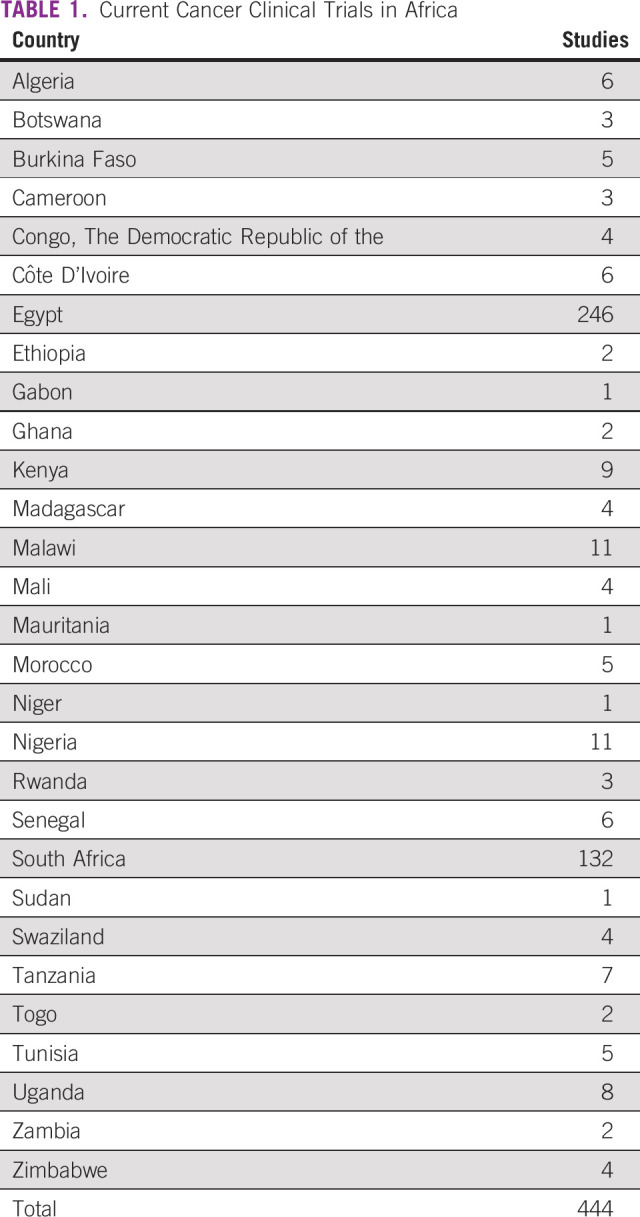
Current Cancer Clinical Trials in Africa

Barriers to conducting cancer-related and other clinical trials in African countries^18-21^ and other low- and middle-income countries include insufficient financial and human capacity, including individuals trained in bioethics for clinical trial protocol evaluation^22,23^; weak regulatory systems, resulting in slow review of clinical trial protocols and new drug applications; lack of supportive research environments; and competing demands on time and resources.^24-27^ Guiding principles for expanding access to clinical trials for patients in Africa,^28^ as well as clinical trial design and implementation considerations in the region,^15,27^ have been outlined, including funding and technical support for local investigator-initiated clinical trials, on the basis of existing needs and resources that have the greatest potential to improve patient care.^14,15,24,29^

To improve awareness about perspectives of cancer research organizations, patient advocacy groups, regulatory agencies, pharmaceutical companies, and global health nonprofit organizations as they relate to expanding cancer clinical trials capacity in Africa (including participation in registration trials)—and promote dialog and joint action among these key stakeholder groups—we convened the satellite session Increasing Diversity, Market Access, and Capacity in Oncology Registration Trials—Is Africa the Answer? at the 2021 Accelerating Anti-Cancer Agent Development and Validation Workshop (Data Supplement). Panelists included three representatives of African research organizations and cancer-focused academic institutions (B.A., J.O., and F.O.); one leader of an African patient advocacy organization (G.N.); three representatives of regulatory agencies, one from Nigeria (M.C.A.) and two from the United States (K.A. and L.F.-A.); two representatives of global pharmaceutical companies (C.K. and J.F.); and two representatives of US-based global health nonprofit organizations involved in health care in Africa (T.N. and J.D.). Clinical oncologists representing US-based oncology professional organizations and academic cancer centers (B.C. and J.G.) moderated the session.

Themes that emerged from the panel presentations and discussion included diversity, equity, and importance of partnerships and collaborations in including African countries in early-stage cancer clinical trials. In this publication, we describe each theme, enumerate associated action items, and outline potential impacts.

## DIVERSITY IN CLINICAL TRIALS IS KEY TO ENSURING CANCER DRUGS ARE EFFECTIVE ACROSS POPULATIONS

Clinical trials are required to determine the safety and efficacy of new products in all patient populations. Participants agreed that the efficacy of standard-of-care cancer drugs in African patients, and patients of African ancestry more broadly, has not been studied sufficiently. Less than 1% of international trials submitted to the US Food and Drug Administration (FDA) for approval of cancer drugs enroll participants from clinical sites in Africa. Most trials that do enroll patients in sub-Saharan Africa typically focus on postmarketing surveillance. Even in the United States, where 20% to 25% of participants in global clinical trials are enrolled, only 2%-4% of participants are of African descent.^30,31^ The representatives of African academic institutions and research organizations emphasized that, on the basis of their clinical experience, the lack of inclusion in registration trials of participants either from Africa or of African descent likely contribute to greater rates of treatment failure and poorer outcomes in African patients.

Panel participants shared their perspectives about reasons for the dearth of clinical trials in sub-Saharan Africa. Representatives of the African Organization for Research and Training in Cancer (AORTIC) and the Uganda Cancer Institute (UCI) described the low priority placed on research, including by country governments, resulting in inadequate funding, insufficient infrastructure (including internet), and insufficient numbers of teams proficient in good clinical practice. Representatives of pharmaceutical companies Bayer (Pittsburgh, PA) and Genentech (South San Francisco, CA) described previous concerns about regulatory infrastructure, data quality and integrity, and patient safety, currently being addressed by these companies through several novel initiatives. From the patient advocacy perspective provided by the leader of the Uganda Women's Cancer Support Organization (UWOCASO, Kampala, Uganda), barriers included lack of understanding among patients about clinical trial procedures and benefits, concerns about drug safety, logistical barriers, gaps in treatment capacity, and insufficient engagement of patient advocates by researchers and regulatory agencies.

Representatives of Nigeria's National Agency for Food and Drug Administration and Control (NAFDAC, Lagos, Nigeria) and FDA discussed the importance of recruiting a diverse population of patients to cancer clinical trials to ensure applicability of results^32^ and noted that sustainable partnerships and collaboration should be the goal of these efforts.

## EQUITY IN ACCESS TO CLINICAL TRIALS AND CANCER CARE PRODUCTS IS A PRIORITY FOR PATIENTS, REGULATORY AGENCIES, CANCER-FOCUSED CIVIL SOCIETY, AND PHARMACEUTICAL COMPANIES

The panelists underscored the importance of ensuring equitable access to clinical trials for patients in Africa.

Panelists from Bayer and Genentech described their commitment to improving equity in cancer care and drew parallels between ensuring access to new medications for African patients and for underserved minority patients in the United States. Examples cited by the panelist from Genentech included a successful effort to recruit US underserved and racial/ethnic minority patients into a phase III clinical trial of its drug tocilizumab for COVID-19 pneumonia by working directly with these communities.^33^ This effort resulted in fast trial accrual and emergency authorization of tocilizumab. Another example was a study of ocrelizumab for multiple sclerosis exclusively among patients of African descent, who are known to have a more severe clinical course and worse outcomes, in both Kenya and the United States.^34^ This successful effort prompted the company to consider additional clinical trials, including in oncology, in sub-Saharan Africa.

Ensuring sustainable research partnerships and long-lasting health care system improvements was a priority for both regulatory agencies and pharmaceutical companies. The panelist from Bayer described conducting needs assessments in Ghana and engaging local experts for prostate cancer capacity building and health care systems strengthening, to ensure sustainable improvements in cancer care. Both the Bayer and Genentech panelists recognized that the considerations for driving equity do not stop after a trial is completed and that plans for market entry and sustained access should be incorporated into research and business strategies.

## MULTILEVEL, MULTISECTORAL COLLABORATION IS KEY TO BRINGING MORE CLINICAL TRIALS TO AFRICA

### Aligning Stakeholder Priorities and Drivers

Panelists agreed that partnerships between African and international stakeholders are critical to expanding high-quality cancer clinical trial activities in Africa. They emphasized the importance of understanding and aligning with priorities and values of key stakeholders in developing and executing collaborations.

AORTIC and UCI representatives strived for improved cancer outcomes for African patients through research and clinical trial capacity building, as well as African investigator-led trials that are both clinically and culturally relevant. They advocated for inclusiveness, collaboration, stronger regulatory frameworks, and Afrocentric approaches.

The patient representative from UWOCASO emphasized the patient advocacy values of improved access to both novel and standard-of-care treatments for patients and inclusion of patient advocates as equal partners in clinical trial planning.

NAFDAC and FDA representatives prioritized ensuring patient health and safety first, followed by data quality, integrity, and applicability to their patients. FDA representatives particularly emphasized compliance with regulations governing clinical trial conduct, including data collection, documentation, and analysis.

Industry representatives highlighted the importance of equitable access, capacity building, and sustainable partnerships. They emphasized the need for quality data, strong regulatory systems, market access, local expertise to facilitate trial implementation, ability to mitigate risk, and need to have a sustainable business model.

The panelists from BIO Ventures for Global Health (BVGH, Seattle, WA) and Bill & Melinda Gates Foundation (BMGF, Seattle, WA) described their support of African countries in bringing high-quality, impactful cancer clinical trials to their patients, including by working together with country regulatory agencies and local investigators.

#### Building on Prior Successes and Lessons Learned

##### HIV/AIDS.

Panelists noted that successful multidisciplinary HIV/AIDS research and clinical care collaborations in African countries—in which robust prevention and treatment programming led to a significant decline in HIV incidence^35^—can serve as models. Capacity and infrastructure developed by the HIV/AIDS community in Africa can be leveraged to build African-led and -driven oncology initiatives, such as UCI (which partners with global institutions including the Fred Hutchinson Cancer Research Center [Fred Hutch]). HIV/AIDS collaborations offer important lessons, including (1) the imperative to focus on humanitarian aims and equity; (2) considering a return on investment that recognizes the positive impact of a low-cost treatment on patient mortality and quality of life; (3) ensuring that clinical trials do not exacerbate existing inequalities in health care access; (4) involving local stakeholders as equal partners in protocol design to avoid skewing research agendas to that of the funders; (5) ensuring that true informed consent is collected and that treatment will be available after the study ends and once the drug is approved; (6) considering and mitigating the effects of brain drain, or exodus of trained personnel, to higher-income countries as a result of cancer clinical trials; (7) avoiding postal research (sending biological samples) and parachute research (sending researchers to conduct short-term projects); and (8) treating African clinical trial experts as equal partners and giving them credit in international meetings and publications.^36,37^

##### Oncology collaborations in sub-Saharan Africa.

Multiple successful initiatives and partnerships that focus on addressing disparities in cancer diagnosis and treatment and increasing access to cancer clinical trials already exist in Africa (Table 2). There are examples of successful building of oncology clinical trial capacity in Nigeria in collaboration with University of Chicago^43^ by the Prostate Cancer Trans-Atlantic Consortium^28^ and by BVGH in several sub-Saharan African countries.^28^ Robust and long-standing collaborations to build local cancer research capacity and improve patient care include the Partners in Health—Rwanda Military Hospital,^44^ University of California, San Francisco—Ocean Road Cancer Institute in Tanzania,^45^ University of Washington/Fred Hutch—UCI,^14^ University of Pennsylvania—Botswana Partnership,^46^ University of North Carolina Chapel Hill—Malawi,^15,16^ and University of Chicago—University of Ibadan, Nigeria.

**TABLE 2:**
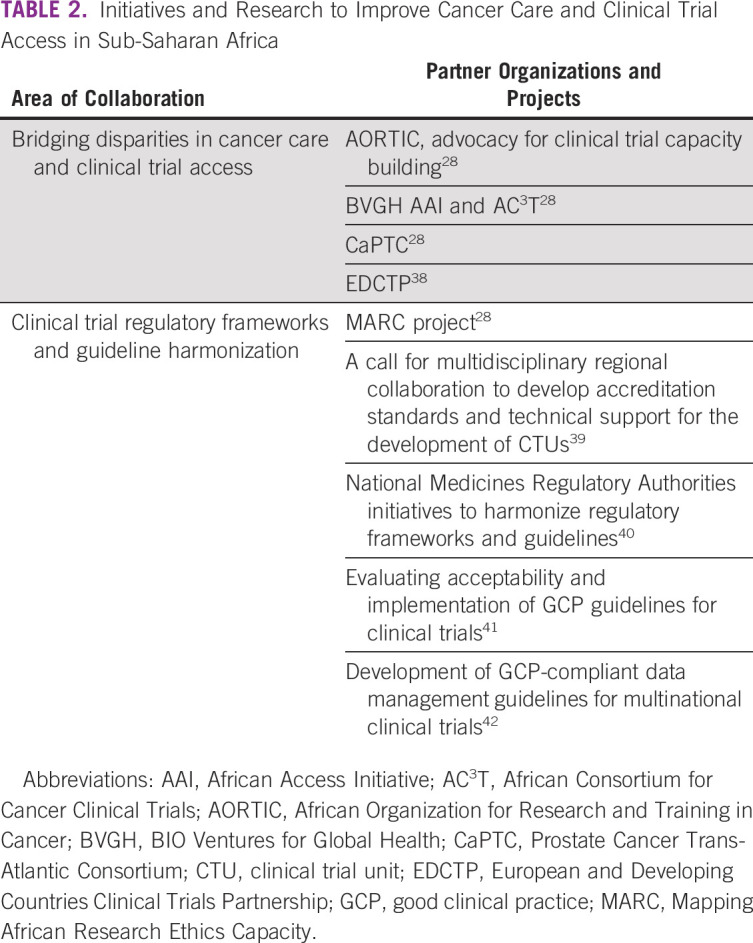
Initiatives and Research to Improve Cancer Care and Clinical Trial Access in Sub-Saharan Africa

### Examples Discussed by Panelists

The panelist from UWOCASO described the key role of patient advocate volunteers in connecting patients to clinical trials at UCI. Such volunteers use their own experience as patients to provide reassurance and accurate information. UWOCASO partners with UCI to provide patient support during treatment and reduce loss to follow-up.

The UCI panelist described the mutually beneficial partnership with Fred Hutch, including in designing and implementing early-stage clinical trials that provided critical insights into treatment of Burkitt's lymphoma.^47^ The UCI-Fred Hutch collaboration provided essential resources for research and clinical trial capacity building and resulted in sustainable investment from the Ugandan Government in cancer care after seeing its success and impact on patient outcomes.

NAFDAC and BMGF have partnered to tailor BMGF's online platform to enable expedited and paperless review of clinical trial protocols in Nigeria.

BVGH has worked on improving access to cancer clinical trials by evaluating and showcasing cancer clinical trial infrastructure and human capacity, as well as the experience and research interests of individual clinical investigators in sub-Saharan Africa.

The Bayer and Genentech panelists emphasized their companies' commitment to corporate responsibility and sustainability. The Genentech panelist shared the company's experience of creating a sub-Saharan Africa leadership team in charge of a 5-year plan to help optimize regulatory and human resource infrastructure, data quality, and patient safety at global sites participating in the company's clinical trials. As a result, in addition to South Africa, the company was able to open cancer clinical trials in Kenya, Uganda, Nigeria, Morocco, and Algeria. A feasibility assessment for a phase I study of lung and colorectal cancer in Kenya is ongoing. The panelist from Genentech also described a coinvestment project with the Government of Côte d'Ivoire that resulted in improved access to cancer drugs for patients and a doubling of sales for the company.

The Bayer panelist shared the company's philosophy about the necessity of assessing a site or country before the initiation of clinical trials, including (1) complete adherence to good clinical practice and ethical protocols; (2) regulatory landscape; (3) market access capability and patterns; and (4) intellectual property protection, legal, and compliance landscapes to ensure patient safety, protection of patient rights, and sustained equitable access to novel therapies. Both Bayer and Genentech emphasized the importance of local experts to advise companies about clinical trial design and information on clinical trial site capabilities, quality, and compliance. Both Bayer and Genentech looked to BVGH, AORTIC, and other global health research and nonprofit organizations to bring key stakeholders together for dialog and collaboration.

The FDA representatives described decades of experience of working with other regulatory agencies around the world to share experiences and data and conduct collaborative reviews^48^ and inspections for marketing applications. They voiced eagerness to collaborate with regulatory agencies in sub-Saharan Africa for the same purpose.

## ACTION ITEMS

Session participants agreed that cancer research institutions, professional organizations, pharmaceutical companies, patient advocacy groups, and global health non-profit organizations need to encourage collaboration through creative partnering models and funding mechanisms that prioritize cross-institutional and cross-country alliances (Table 3).^36^ In addition to expanding access to care, such partnerships should focus on building both clinical trial and standard-of-care treatment capacity, empowering more African patients to participate in trials by partnering with patient advocates, and strengthening African regulatory agencies.

**TABLE 3:**
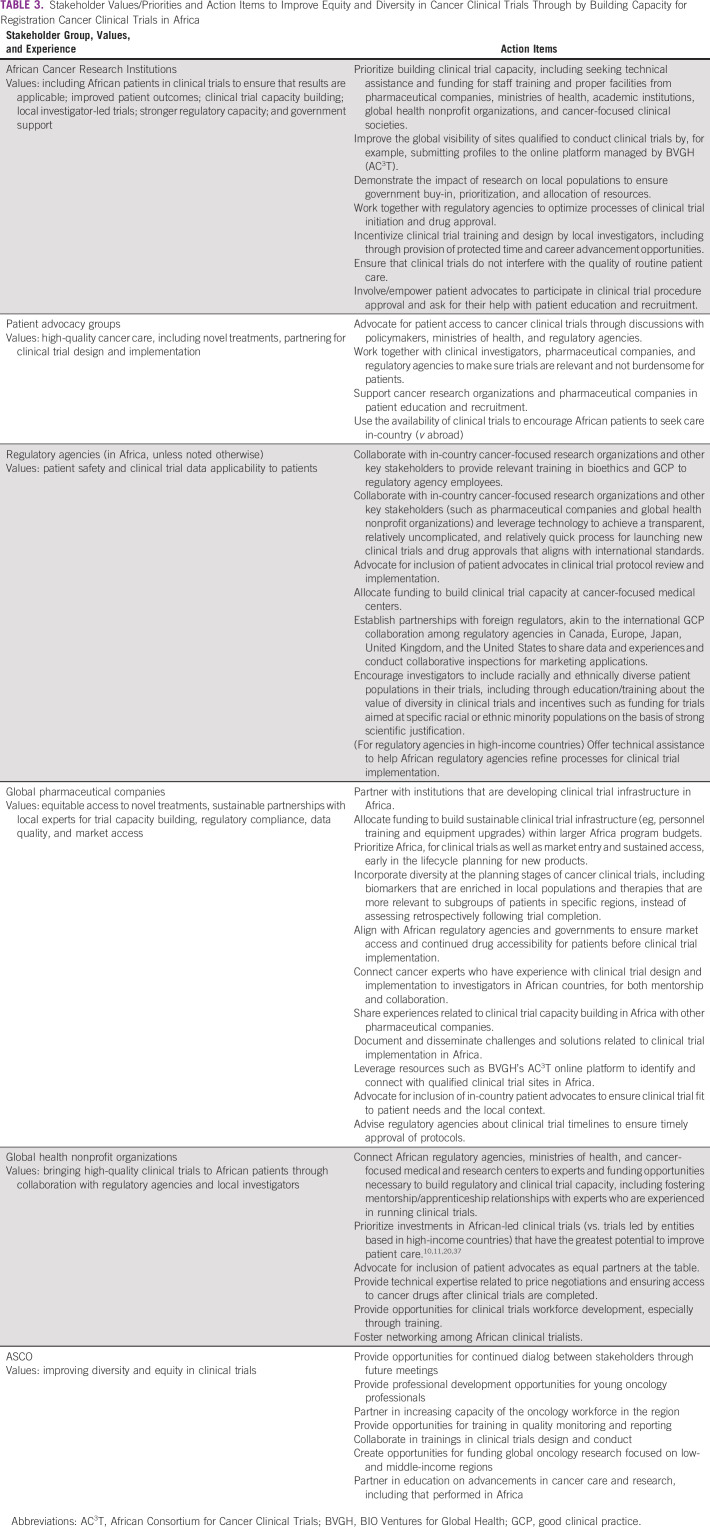
Stakeholder Values/Priorities and Action Items to Improve Equity and Diversity in Cancer Clinical Trials Through by Building Capacity for Registration Cancer Clinical Trials in Africa

The ASCO and the American Cancer Society, organizers and moderators of this Accelerating Anti-Cancer Agent Development and Validation satellite session, have a strong commitment to achieving diversity and equity in cancer clinical trials. Using their name recognition and convening power, these organizations were able to stimulate dialog on an international scale through this panel. These organizations are leveraging their influence, networks, and convening power to stimulate dialog across sectors and international borders, including through this panel session. Together, they are committing to continue the momentum for building a robust clinical trials infrastructure in sub-Saharan Africa, through partnerships with African cancer institutions, professional societies, cancer clinicians, researchers, patient advocates, industry, regulatory agencies, and all stakeholders.

Action items for each group of stakeholders were based on each group's values and priorities and focused on improving diversity and equity in registration cancer clinical trials by building clinical trial capacity in Africa.

African cancer research institutions will collaborate with key stakeholders to lead: (1) building clinical trial capacity, including by incentivizing clinical trial training of and design by local investigators and working with patient advocates to promote patient education and recruitment; (2) working with regulatory agencies to optimize clinical trial initiation and drug approval; (3) ensuring government buy-in by showing impact of clinical trials on patient outcomes; and (4) integrating clinical trial processes into high-quality patient care.

Patient advocacy groups will play an essential role in obtaining buy-in for clinical trial participation from patients and advocating for resources for clinical trial capacity building with key stakeholders.

Regulatory agencies in Africa will collaborate with other key stakeholders to streamline clinical trial opening and drug approval and ensure data integrity and applicability. The agencies will also encourage pharmaceutical companies and investigators to devise strategies to include racially and ethnically diverse patients, as well as underserved patients, in their trials.

Global pharmaceutical companies will focus on: (1) building clinical trial capacity in Africa and including patient advocates early to ensure that trials are appropriate to the local context; (2) prioritizing Africa for clinical trials and considering diversity at the planning stages of clinical trials; (3) connecting cancer clinical trial experts to African clinical investigators for mentorship; and (4) working with country regulatory agencies to meet trial timelines and with governments to ensure drug accessibility and market access after drug approval.

Global health nonprofit organizations will connect key stakeholders to funding, training opportunities, and mentors for building clinical trial capacity, including by prioritizing investment in African investigator-led trials.^10,11,20,37^ They will also lend their expertise in price negotiation to ensure drug access after trial ending.

ASCO will (1) facilitate dialog and collaboration of key stakeholders; (2) collaborate to increase clinical trial capacity in the region, including through training; (3) create funding opportunities for cancer research in low- and middle-income countries; and (4) create professional development opportunities and cancer care and research education opportunities, including in Africa.

## ANTICIPATED IMPACTS

Achievement of the proposed action items will lead to improved access for African patients to locally relevant investigator-initiated clinical trials and to registered products with safety and efficacy that have been validated in African populations, resulting in better clinical outcomes. The knowledge and experience gained in improving equitable access to novel drugs in underserved settings can be leveraged to do the same for underserved patients in high-income countries.

In conclusion, diversity, market access, and capacity in oncology registration trials can be improved by implementing trials in Africa in partnership with local investigators, patient advocates, and regulatory agencies. Our panel discussion about strategies for improving diversity and equity in clinical trials, which were priorities for all panelists, resulted in valuable insights about the values and priorities of key stakeholders related to bringing early-stage clinical trials to Africa. The discussion generated specific action items for each group of stakeholders on the basis of their priorities, that, once implemented, will result in increased applicability of trial data to broader groups of patients, more equitable care, and improved patient outcomes in Africa and globally.
