# Erratum

**DOI:** 10.1200/GO.22.00230

**Published:** 2022-08-23

The article by Kizub et al entitled “Action for Increasing Diversity, Market Access, and Capacity in Oncology Registration Trials—Is Africa the Answer? Report From a Satellite Session of the Accelerating Anti-Cancer Agent Development and Validation Workshop” (*JCO Glob Oncol* 10.1200/GO.22.00117) was published online June 17, 2022, with an error.

In the author list, “Jamie Freedman, PhD^13^” should have read, “Jamie Freedman, **MD,** PhD^13.^”

This has been corrected as of August 23, 2022. *JCO Global Oncology* apologizes for the error.

